# Orlando Magic: report from the 57th meeting of the American Society of Haematology, 5–7 December 2015, Orlando, USA

**DOI:** 10.3332/ecancer.2016.612

**Published:** 2016-01-14

**Authors:** Luca Mazzarella

**Affiliations:** European Institute of Oncology, Via Ripamonti 435, Milan 20141, Italy

**Keywords:** myeloma, lymphoma, leukemia

## Abstract

The 57th American Society of Haematology (ASH) meeting held in Orlando, FL was certainly the year when myeloma management changed for good, with a plethora of newly Food and Drug Administration (FDA)-approved drugs showing impressive outcome improvements and the introduction of new techniques for disease monitoring. Also, chimeric antigen receptor (CAR) T cells continued their triumphal march, consolidating their success in lymphoma and chronic lymhocytic leukaemia (CLL) and venturing into new fields such as again multiple myeloma. Some experimental drugs showed long-awaited results (midostaurin in FLT3-mutated acute myeloid leukaemia (AML)) and some brand new drugs showed promising results in the clinic after extensive preclinical studies, such as those targeting new epigenetic factors (histone methyltransferases) and apoptosis.

## Myeloid neoplasms

The long-awaited results of the RATIFY trial testing **midostaurin** for FLT3-mutated AML patients were presented (abst #6). It is worth remembering that AML is possibly one of the few fields in oncology where no targeted therapy is yet approved and the mainstay of treatment remains classic chemotherapy. In the RATIFY trial, adult AML patients <60 years old were tested for FLT3 mutations, and if found positive were randomised to adding placebo or the FLT3 inhibitor midostaurin to standard chemotherapy during the entire treatment course (induction with daunorubicin+ Ara-C, consolidation with Ara-C and maintenance with midostaurin/placebo). Midostaurin provided a significant benefit in the four year overall survival (OS) (51.4% versus 44.2%, hazard ratio (HR) 0.77, *p* = 0.0074) and a modest improvement in complete remission (CR) rate (66% versus 59%), but only if post-consolidation responses were included. The results were somewhat polluted by a higher rate of transplant in the midostaurin arm, for reasons not entirely clear, but even after correcting for this, the OS increment was significant. Incidentally, the same group also consolidated their previous investigations in a role for sorafenib in improving post-transplant relapse rate and survival in FLT3-mutated patients (abst #864). An important point raised by the lead investigator, Dr Richard Stone, is that the benefit for midostaurin in RATIFY was observed in all FLT3 mutation subgroups, even those with the D835 mutation, now known to be biologically and pharmacologically distinct from the more common internal tandem duplications (ITD). This led the author to speculate that midostaurin’s poor specificity against FLT3 (it was initially developed as a vascular endothelial growth factor (VEGF) inhibitor) might have caused the inhibition of other relevant pathways and thereby might make it effective also in non FLT3-mutated AMLs. Thus,despite the prevalent tendency for preferring highly specific inhibitors in targeted therapies, the pleiotropic inhibition allowed by nonspecific inhibitors might play favourably in some circumstances. On the other hand, another study with a very specific FLT3-targeting agent, ASP2215, showed promising responses selectively in FLT3-ITD AMLs in a phase 1–2 trial (abst #321). Maybe we are about to witness some success in the targeting of FLT3-mutated AMLs, which we know is a poor prognosis subgroup with a dismal record of killing several targeted agents when tested in advanced clinical trials.

Particularly intriguing were the results presented by Dr Chun Wei-Lee on therapeutic targeting of splicing in leukaemia (abst #4). The authors elegantly demonstrated that mutations affecting splicing factors in AML and myelodysplastic syndrome (MDS) induce very specific transcriptional alterations that probably contribute to leukaemia development. This is a hot topic that was extensively covered in several sessions on the basic biology of MDS and AML (abst #139–143). Dr Lee’s study in particular stands out: realising that these mutations are mutually exclusive and mostly heterozygous, they showed that mutated cells cannot tolerate further reduction of splicing activity and are selectively susceptible to spliceosome inhibitors like E7107 from H3 biomedicine. These results have likely opened a promising new field for therapy development.

Direct or indirect regulators of chromatin have attracted considerable interest in recent years, and advanced or definitive data on early phase trials were presented for drugs targeting IDH1/2 and hDOT1L. Dr Stein (#323) presented a phase 1/2 dose-escalation and expansion study of the isocitrate dehydrogenase (IDH inhibitor) AG-221 in patients with advanced haematologic malignancies (mostly relapsed/refractory AMLs). The results were very promising with an overall response rate (ORR) of 41% and a good toxicity profile, without an identifiable maximum tolerated dose (MTD) within the tested range. Dr Armstrong presented an updated data on phase I study of H3K79 inhibitor pinometostat (EPZ5676) in relapsed/refractory myeloid neoplasms (with expansion cohorts in mixed lineage leukaemia (MLL)-aberrant AMLs) (abst #2547). The toxicity profile was very good and an MTD was not achieved. As shown in previous ASH meetings, the response to this drug is dominated by a strong induction of neutrophilic differentiation of leukaemic cells. The ORR was 25%, with only two CR observed, but with very limited toxicity. An analogous trial on paediatric leukaemias is ongoing (#3792).

Perhaps less innovative but of great importance in clinical routine was the prospective comparison of reduced intensity (RIC) versus myeloablative (MC) conditioning regimens for allo-transplant in MDS and AML. RIC has been shown to be better tolerated and has been proposed as a viable alternative to MC, but doubts have arisen with regards to its efficacy. The study presented by Dr Scott (LBA-8), which was stopped for inferiority after a median of 18 months follow-up, showed that overall RIC was associated with a higher incidence of relapse (48% versus 13%, *p* < 0.01) and a trend towards poorer OS (67% versus 77%, *p* = not significant (NS)). It has to be pointed out that at subgroup analysis, the difference was pretty clear for AML but much less striking for MDS, leaving the jury still wondering as to whether MC should be abandoned in the MDS setting where the smaller incidence of relapse and usually more advanced age might still lead clinicians to opt for a lesser toxic treatment.

## Lymphoid neoplasms

### Minimal residual disease (MRD) measurement

In several diseases, recent therapeutic regimens produce very elevated rates of CR as defined by conventional assays in the bone marrow and peripheral blood [1]. Still a majority of patients relapse, clearly emphasizing the need for more sensitive measurements of minimal residual disease (MRD) to predict outcome and stratify risk for best treatment intensity. This approach is consolidated in AML, where polymerase chain reaction (PCR)-based strategies have been employed for a long time and next generation sequencing (NGS)-based techniques are increasingly employed, as also shown at this ASH (for DNMT3 mutations #226, for nucleophosmin NPM mutations #227). In lymphoid diseases, the distinctive clonality of lymphocyte populations can be appreciated by quantifying immunoglobulin or T Cell receptor rearrangements through dedicated PCR or NGS-based assays, as in abst #191, where a NGS-based rearrangement detection was retrospectively applied to the IFM/DFCI 2009 trial and showed predictive power, discriminating between early and late relapse in patients with conventionally defined CR. In addition, new generation cytometry techniques like 8-color flow cytometry or mass cytometry achieve higher sensitivity and increased prognostic power (abst #19, #191, #367) compared to 4/6 color flow cytometry; head-to-head comparisons between different second generation techniques are still ongoing. In lymphoma, several DNA-based MRD detection strategies showed prognostic power: a PCR-based immunoglobulin rearrangement detection in mantle cell lymphoma (abst #338) and a CAPP-seq based strategy [2] targeting frequent somatic mutations in diffuse large B-cell lymhoma (DLBCL) (abst #114).

### Experimental drugs in myeloma

This was a big year for multiple myeloma (MM). Several new drugs have been recently granted FDA approval, and CAR T cells have found their way into this disease with impressive results. Advancements in myeloma were celebrated with the presentation of the Ernest Beutler award to Goldberg and Richardson for the development of protease inhibitors.

In the weeks preceding ASH, three new drugs targeting myeloma were approved by FDA. Several updates on ongoing studies with different combinations of investigational drugs with established regimens were presented.

Elotuzumab, an immunostimulatory monoclonal antibody that recognises signalling lymphocytic activation molecule F7 (SLAM F7), showed about 30% improvement in progression-free survival (PFS), and preliminarily in OS when added to either lenalidomide/dexamethasone (19.4 versus 14.9 months, HR 0.73 for PFS, 43.7 months compared with a median of 39.6, HR = 0.77 for OS in the ELOQUENT-2 study, abst #28) or to bortezomib and dexamethasone (HR 0.7 for both PFS and OS in the study by Palumbo *et al*, abst #510) in relapsed/refractory multiple myeloma (RRMM).

Daratumumab elicits both direct and immune-mediated killing by targeting CD38, a molecule expressed on both myeloma cells and lymphocytes. Additionally the drug counteracts the anti-inflammatory effect elicited by the ectoenzyme activity of CD38 which causes adenosine-induced T-cell anergy. Daratuzumab showed efficacy with lenalidomide/dexamethasone in RRMM (phase I/II GEN503 study, abst #507). There was a 81% ORR, with responses achieved very rapidly (one month was the median time to first response); the PFS and OS medians had not yet been achieved after 18 months of median follow-up. Toxicity was very common but did not seem to be increased over that caused by lenalidomide/dexamethasone. Interestingly, daratumumab was also studied in RRMM as monotherapy in the GEN501 and SIRIUS trials, of which a combined analysis was presented (abst #29). Even if the ORR was only 13%, the drug appears to provide significant survival benefit with a median OS of 19.9 months across all studies and strata. Among responders, a median OS was not reached, whereas in those who had minimal or no response, OS was 17.5 months, which favourably compares with historical controls. Therefore, it can be considered in patients who have exhausted all other therapeutic options.

Ixazomib, a new generation reversible protease inhibitor, showed a 5.9 month improvement in PFS (20.6 versus 14.7 months, HR 0.742) when added to lenalidomide-dexamethasone in RRMM (abst #727). All genetic subgroups benefitted from the addition, including those with the unfavourable del (17). Given the favourable toxicity profile that matched that of earlier phase studies [3] and good manageability, this all-oral chemo-free regimen is very promising. The drug is being tested in the first line too, either with lenalidomide and dexamethasone (TOURMALINE-MM2 study) or with cyclophosphamide and dexamethasone where it showed an ORR of 71% (abst #26).

It is difficult to foresee which of these drugs is going to take the lead in the future therapy of MM; roughly 30% improvement in OS seem to be common across all studies in RRMM, despite differences in the study populations. Head-to-head comparisons will be required, but we can safely state that new standards in myeloma therapy have been affirmed at this ASH.

### Immune checkpoint inhibitors in myeloma and lymphoma

The anti-PDL1 pembrolizumab is finding its way into myeloma treatment, after success in lung cancer. In the KEYNOTE-023phase 1 trial, pembrolizumab was tested in combination with lenalidomide and low-dose dexamethasone. A recommended fixed dose of 200 mg was identified and a good ORR of 76% was observed with durable responses (abst #505). Pembrolizumab is also being studied with pomalidomide and dexamethasone (abst #506) with less definitive results (ORR of 50%). Preliminary results were also shown in a phase 1 trial on relapsed/refractory primary mediastinal large B-cell lymphoma (abst #3986)

### CAR T cells

One of the ASH highlights was certainly the impressive preliminary results shown by Dr Kocherferder on the new B-cell maturation antigen (BCMA) CAR T cells (LBA #1) in myeloma. In this dose-escalating phase 1 trial enrolling heavily pretreated patients, one of the two patients receiving the highest dose showed a complete and durable remission (ongoing after 14 weeks) from a baseline marrow infiltration of 90%. The price to pay was an acute cytokine syndrome that required ICU management, similar to that observed in other contexts. Partial remissions (PR) were observed also at lower doses.

In lymphoma, CAR T cells have now been around for a couple of years [4], and we have seen consolidated data across different lymphoma subtypes especially with CARs against the most popular target to date, CD19. ORR ranged from 47% in DLBCL, 73% in follicular lymphoma, and even 100% (of nine patients) in CLL (abstracts #183 and #184). So far, most CAR trials have concentrated on the setting of relapsed/refractory lymphoma, and proposed as an effective means to obtain durable response to bring patients to allogeneic transplantation. Dr Kochenderfer now showed promising results also in patients relapsing after allo-transplant, in which a CR rate of 30% was achieved with a single anti-CD19 CAR T cell infusion with no chemotherapy (abst #99). These regimens are still affected by severe toxicity and heterogeneity of autologous T cell expansions, so important points to define are the best lymphodepletion conditioning regimen and the best T cell subpopulation to use. The Seattle group showed that the addition of fludarabine to cyclophosphamide may improve T cell expansion (abst #184).

### Experimental drugs in lymphoma and CLL

After the dramatic advancements presented at past ASH conferences, this year we saw the consolidation of kinase inhibitors in CLL and lymphoma.

Idelalisib was tested against placebo in combination with standard bendamustine and rituximab in a randomised phase 3 study presented by Dr Zelentz (LBA #5). The study was unblinded at the second interim analysis because of the overwhelming superiority of the idelalisib combination with a PFS of 23.1 versus 11.1 months (HR 0.33, *p* < 0.0001) despite a more modest difference in ORR (68% versus 45%). The preliminary OS analysis also showed benefit (HR 0.55, *p* = 0.008); however subgroup analysis showed that patients with disease refractory to several prior lines also had less benefit from the idelalisib addition.

The role of the Bruton tyrosine kinase ibrutinib in lymphoma was consolidated by several studies. Ibrutinib demonstrated superiority over another investigational drug, temsirolimus, in a head-to head comparison in mantle cell lymphoma in second line (abst #469). It was also tested as a first line treatment in follicular lymphoma, where it showed good responses in a phase 2 trial in combination with rituximab (82% ORR of which 27% CR, abst #470) and in a phase 1 trial in combination with rituximab and lenalidomide (91% ORR); the toxicity was manageable without dose limiting toxicities (DLT) identification, although an unexpectedly elevated rate of severe rash was observed (abst #471). Ibrutinib also showed efficacy in difficult-to-treat primary central nervous system lymphomas, when combined with systemic and intrathecal chemotherapy (abst #472).

In CLL, where ibrutinib was FDA-approved first, the new randomised phase 3 trial against chlorambucil presented by Tedeschi *et al* (abst #495, recently published [5]) confirmed the status of ibrutinib as the new standard of care also in the first line treatment of elderly patients. Superiority was observed both in terms of PFS (median not reached [NR] versus 18.9 months; HR 0.16) and OS (median was not reached in either arm, HR 0.16, 24-month OS rates were 98% versus 85%). It is to be noted that high-risk patients such as del 17 carriers were excluded from the study.

Among drugs with radically new mechanisms of actions, two stood out: BCL2 agonists and EZH2 inhibitors.

Dr Stilgenbauer (#LBA5) presented very intriguing results on the BCL2 agonist venetoclax, for which the phase 1 toxicity study was recently completed without the identification of an MTD [6]. The presented phase 2, single arm study was restricted to CLL patients with the very unfavourable 17p deletion. The CR rate was around 10% and partial responses (PR) were at 69%, but almost all patients achieved normalisation of peripheral blood lymphocyte counts. Tumour lysis syndrome was very common but its clinical significance was greatly minimised through the adoption of a stepwise weekly dose increase to the target of 400 mg daily. Venetoclax is sure to exert a significant impact on this class of patients with dismal prognosis.

The EZH2 inhibitor tazemetostat was tested in the first-in-human dose-escalating phase 1 trial enrolling both solid and haematological tumour patients (abst #471). Among which 16 patients had non-Hodgkin’s lymphoma and were separately evaluated. A total of nine patients had an objective response, and the treatment was overall well tolerated with four out of 55 total patients experiencing grade 3–4 events (hypertension, liver toxicity, thrombocytopaenia, neutropaenia).
